# The Influence of Parents’ Nutritional Education Program on Their Infants’ Metabolic Health

**DOI:** 10.3390/nu14132671

**Published:** 2022-06-28

**Authors:** Dagmara Woźniak, Tomasz Podgórski, Małgorzata Dobrzyńska, Juliusz Przysławski, Sylwia Drzymała, Sławomira Drzymała-Czyż

**Affiliations:** 1 Department of Bromatology, Faculty of Pharmacy, Poznan University of Medical Sciences, Rokietnicka 3 Street, 60-806 Poznań, Poland; dagmara.wozniak@student.ump.edu.pl (D.W.); mdobrzynska@ump.edu.pl (M.D.); jotespe@ump.edu.pl (J.P.); 2 Doctoral School, Faculty of Pharmacy, Poznan University of Medical Sciences, Fredry 10 Street, 61-701 Poznań, Poland; 3 Department of Physiology and Biochemistry, Poznan University of Physical Education, Królowej Jadwigi 27/39, 61-871 Poznań, Poland; podgorski@awf.poznan.pl; 4 Department of Pediatric Gastroenterology and Metabolic Diseases, Poznan University of Medical Sciences, Szpitalna 27/33, 60-572 Poznań, Poland; sylwia-drzymala@wp.pl

**Keywords:** child nutrition, development, early nutrition, nutritional programming, Z-score, BMI, weight

## Abstract

Childhood obesity is considered an epidemic in both developing and developed countries. Children obesity plays a vital role in children’s development and has a profound impact on their health in adult life. Although the etiology of obesity is multifactorial, it can be prevented. According to research, feeding practices, developing eating habits, and parenting styles are of primary importance. Despite the widespread access to information on children’s nutrition, parents still make many mistakes preparing their meals. Thus, this study aimed to evaluate the impact of parents’ nutritional education on children’s selected anthropometric-metabolic parameters during their first year of life. The study comprised a group of parents of 203 Polish infants. Their parents were randomly assigned to one of two groups: the intervention group received intensive mobile nutritional education for a year, while the control group received no intervention. Blood tests and anthropometric measures were performed on both groups at the beginning of the study and one year later. Our study showed that parental nutritional education influences, among others. the BMI Z-score (the difference between the groups was 1.039) and the TG/HDL ratio (*p* < 0.001) in children. The final results of our study showed that proper nutritional education could improve children’s nutritional status at the population level.

## 1. Introduction

Childhood obesity is considered an epidemic in both developing and developed countries [1]. To our greatest concern, it is an omnipresent phenomenon throughout Europe. According to experts, 90% of children with obesity suffer from simple obesity, which is caused by an excess of calories and simple sugars [2].

Obesity in children plays a vital role in their development and profoundly impacts their health in adult life [3]. It is connected with numerous health problems, such as cardiovascular diseases, diabetes, and other endocrine disorders [1,2,3]. Children with obesity are more likely to become adults with obesity [1,3]. Obesity decreases the quality of life, reduces life expectancy and strongly affects psychological health [1,2,3].

Although the etiology of obesity is multifactorial, it can be prevented [3,4,5]. According to research, feeding practices, developing eating habits and parenting styles are of primary importance [6,7,8,9,10,11]. They are also considered as modifiable determinants of children’s weight status and eating behaviors [6]. Other predicting obesity factors include parents with obesity, poor socioeconomic status, low nutritional education knowledge, poor nutritional behaviors, and family habits [6,7,8,9,10,11]. Unhealthy eating habits are developed during infancy [7]. During the first years of children’s lives, parents control their dietary intake, reactions to new products, and food attitude [9]. Thus, it is crucial for parents to acquire proper nutritional knowledge. In parents’ understanding of infant hunger and satiety cues and the rules of healthy eating mainly lies child’s total energy intake [10,11]. It is also essential to develop children’s accurate responsiveness to hunger and satiety and shape correct eating habits by providing them with the right amount of food in the right time [10,11].

Besides nutritional knowledge, other factors affect the nutritional status of children. Moreover, the level of parents’ education has been proven to affect children’s weight [7,12,13,14]. Several studies showed the effectiveness of parental nutritional education in improving their children’s health [15,16,17]. The concepts of nutritional programming focus on the influence of parents, especially mothers, on the nutritional status of the child in the youngest years.

Nutritional programming explains that both an excess and a deficiency of nutrients in the first 1000 days of a child can permanently exert effects upon developing tissues and reprogram the metabolism [18,19,20]. This has a crucial impact on health in adulthood [18,19,20]. Nutritional programming evaluates the correlation between children’s malnutrition and the prevalence of cardiovascular disease, osteoporosis, obesity, hypertension, insulin resistance, impaired glucose tolerance, and type 2 diabetes in adult life [21,22,23,24,25]. Despite the widespread access to information on children’s nutrition, parents still make many mistakes preparing their meals. According to PITNUTS study, parents expanded the diet of their babies too soon and gave their children too many meals, especially snacks [26]. Children’s diets were too high in energy, simple sugars, and protein, and low in long chain polyunsaturated fatty acids (LCPUFA), fats, vitamin D, vitamin E, potassium, calcium, and fiber [26]. Therefore, their diet was deficient in both macro- and micronutrients. Since proper nutrition is one of the most important factors in early childhood development, which profoundly impacts child’s susceptibility to many diseases. Proper nutrition of children cannot be ensured without an adequate level of nutritional knowledge of the parents. 

Thus, this study aimed to evaluate the impact of parents’ nutritional education on infants’ selected anthropometric-metabolic parameters during their first year of life. The research hypothesis assumes that nutritional intervention significantly improves metabolic and anthropometric parameters in infants, with particular emphasis on body weight. If proven to be significant, the results may trigger the development of novel approaches to the field of childhood nutrition that could improve children’s nutritional status at the population level.

## 2. Materials and Methods

### 2.1. Participants

The study comprised a group of 203 Polish infants and their parents. Out of 203 infants, 44% (*n* = 89) were males and 56% (*n* = 114) females. Both parents participated in the research unless indicated otherwise (the other parent’s lack, the other parent’s absence, etc.). Infants were recruited to the study at the first vaccination schedule between the ages of six and eight weeks. In Poland, infant immunization is compulsory and occurs in the Pediatric Outpatient Clinics. The study was conducted in five Paediatric Outpatient Clinics in Poland during 2019–2022. The Outpatient Clinics were public health providers and were selected according to the region’s highest patient population.

Parents of 160 children completed the study. Parents of nine children were lost to follow-up, while parents of thirteen children failed to appropriately complete the nutrition diary for their children, and twenty-one children were supplied with dietary supplements (mostly iron) during the study; thus, their blood parameters were not included in the statistical analyses.

Inclusion criteria were: children born between the 36th and 42nd week of pregnancy, children less than eight weeks of age, written consent from children’s parent or legal guardian. Each child received a minimum Apgar score of 8 at birth. 

Exclusion criteria: children’s birth weight below 2500 g, history of chronic systemic disease, gastrointestinal diseases which result in digestion and absorption disorders and other severe systemic diseases (cancer, endocrinopathies, connective tissue diseases, kidney diseases, diabetes).

### 2.2. Study Intervention

Parents were randomly assigned to one of two subgroups. For around a year, parents of 102 infants received intensive mobile nutritional education. The planned intervention involved intensive nutritional education delivered to parents via short text messages about their infants’ nutrition (approximately four to six times a week). Text messages’ content was adjusted to a few conditions (e.g., infant’s age, stage of development or season of the year). The parents of the other 101 infants served as the control group.

### 2.3. Study Measurements

Both at the beginning of the study and after the intervention, the anthropometric (body weight, body length, body mass index—BMI) and metabolic (cholesterol, triglycerides—TG, HDL—high-density lipoprotein, LDL—low-density lipoprotein, glucose, protein, albumins) parameters in children were examined.

We also analyzed parents’ age, place of residents and level of education. When it comes to age, we calculated the median and 1st and 3rd quartiles. Place of residents was analyzed according to criteria: village (from the city agglomeration—in the vicinity of Poznań, a city with fewer than 500 thousand residents, a city with more than 500 thousand residents) and the education criteria were as follows: primary (primary school), secondary (high-school), higher (university degree).

### 2.4. Statistical Analyses

The primary endpoint was as follows: Z-score for BMI and the difference of Z-score for BMI throughout the study between the groups.

Power analyses were carried out using G*Power v. 3 (University of Dusseldorf, Dusseldorf, Germany). Assuming the 80% power of the test and the significance level of 5% (two-tailed), detection of the expected effect (the absolute difference between mean BMI Z-scores in the two groups ≥ 0.45) necessitates data from 158 patients. The predicted percentage of patients lost to follow-up is 20%, yielding the final group size of 198 children. Therefore, 203 children will be recruited and randomized.

Obtained data were analyzed using MedCalc 19.6 (MedCalc Software, Ostend, Belgium, 1993–2020) and GraphPadPrism 5.01 (GraphPad Software, Inc., La Jolla, CA, USA) statistical software. For all parameters, medians, and 1st–3rd quartiles were calculated unless indicated otherwise (for the data of normal distribution mean, standard deviation—SD and 1st–3rd quartiles were calculated). The Shapiro-Wilk test was used to check the normality of the data distribution. Statistical differences between groups were tested using Mann-Whitney, Student’s and Chi^2^ tests. A *p*-value of <0.05 was considered statistically significant. Statistical analyses were conducted on the basis of descriptive statistics. The nutritional status of the children was evaluated based on the standardized Z-score for BMI concerning the cut-off points established by the WHO [27]. The standard BMI falls within the range of the Z-score from −2 SD to 1 SD, underweight: <−2 SD to −3 SD, overweight: 1 SD to <2 SD, obesity from 2 SD to 3 SD. TG/HDL ratio was calculated for every child. The cut-off point of 2.0 was established [28,29].

### 2.5. Ethical Consideration

All subjects gave their informed consent for inclusion before participating in the study. Parents were asked to read the informed consent form and consent to collect and process personal data form. Then they were asked to consent to the participation and data processing. The research was conducted in accordance with the Declaration of Helsinki, and the Bioethical Committee approved the protocol of the Poznan University of Medical Sciences in Poznań, Poland (decision no. 723/19).

### 2.6. Blood Collection

The capillary blood was collected by professional, qualified laboratory personnel. All safety precautions were strictly followed during blood collection. Blood samples were obtained from heel-prick using a disposable Medlance^®^ Red lancet-spike (HTL-Zone, Berlin, Germany) with a 1.5-mm blade and 2.0-mm penetration depth. Approximately 300 μL of capillary blood was collected into a Microvette^®^ CB 300 tube (Sarstedt, Nümbrect, Germany) containing K2-EDTA (EDTA dipotassium salt) as anticoagulant for plasma lipid profile measurement. Additionally, 300 μL of capillary blood was collected in a Microvette^®^ CB 300 Z tube (Sarstedt, Nümbrect, Germany) with a clotting activator.

Glucose, albumin and total protein measurements were made in the obtained serum (separation by centrifugation 1500× *g*, 4 °C, 10 min).

### 2.7. Biochemical Measurements

Triglycerides; TG (ACCENT-200 TG, Cormay, Cat No. 7-253, Łomianki Poland), total cholesterol; TC (ACCENT-200 CHOL, Cormay, Cat No. 7-204, Łomianki Poland), high-density lipoprotein cholesterol; HDL (ACCENT-200 HDL DIRECT II GENERATION, Cormay, Cat No. 7-279, Łomianki Poland) and low-density lipoprotein cholesterol; LDL (ACCENT-200 LDL DIRECT II GENERATION, Cormay, Cat No. 7-280, Łomianki Poland) concentrations were measured as an enzymatic, colorimetric reactions. Furthermore, within freshly separated serum, the concentration of glucose; GLU (ACCENT-200 GLUCOSE, Cormay, Cat No. 7-201, Łomianki Poland), albumin; ALB (ACCENT-200 ALBUMIN, Cormay, Cat No. 7-238, Łomianki Poland) and total protein; TP (ACCENT-200 TOTAL PROTEIN II GENERATION, Cormay, Cat No. 7-236, Łomianki Poland) were measured using colorimetric methods. All biochemical parameters were determined using of the Accent 220S (Cormay, Łomianki, Poland) biochemical analyser. The sensitivity of the methods for serum TG, TC, HDL, LDL, GLU, ALB and TP were 5.7 mg/dL, 2.85 mg/dL, 0.5 mg/dL, 2.0 mg/dL, 2.8 mg/dL, 11.4 g/L and 1.5 g/L, respectively. The coefficients of variation (CVs) for the TG assay were 1.29% and 2.15%, TC were 1.61% and 3.03%, HDL were 1.69% and 1.40%, LDL were 2.12% and 2.23%, GLU were 0.71% and 2.38%, ALB were 0.96% and 0.94% and TP were 0.59% and 1.50%, respectively, to the repeatability- and reproducibility-assay CVs.

Additionally, the Friedewald equation [TC (mg/dL) − HDL (mg/dL) − TG (mg/dL)/5] was used for calculated LDL concentration (mg/dL) assessment [30].

### 2.8. Anthropometric Measurements and Dietary Intake

At the beginning and at the end of the study, the nurse measured the children with a pediatric stadiometer and weighed them with a pediatric scale.

Parents provided information about their children’s menu over an average three-day period, including meals, snacks, and fluids. Infants’ menus also contained information on breastfeeding and formula milk. Parents accounted for the amount of milk drunk by the infants. Breastfeeding and formula feeding were taken into account in calculating quantitative and qualitative dietary intake. The eating schedule had to include one weekend day. Parents were trained by a certified dietitian and instructed on how to complete the food diary. The instruction contained hints on filling in the food diary correctly and a link to the page to help determine the proper food portions (www.ilewazy.pl). A contact (e-mail and telephone number) to the dietitian was also available in case of any questions. 

Children’s diets were analyzed using Dietetyk 2015 (Jumar Software, Poznan, Poland). The NutritionData.com database was applied in the study. According to Polish nutritional standards, the average daily macronutrient intake of children was calculated and compared to the recommended dietary allowance (RDA) [31]. The dietary supplementation was not included in the assessment.

## 3. Results

The majority of responders lived in the village from the city agglomeration (60%) and held a university degree (76%). Table 1 presents the characteristics of the groups.

Table 2 presents birth weight in children at the beginning and at the end of the study. The difference in mean Z-score for BMI between the groups was 1.039. At the baseline, the groups did not differ when it comes to birth weight, Z-score for birth weight and Z-score for BMI. However, at the end of the study, the intervention group has significantly lower weight and BMI comparing to control group. As mentioned before, the standard BMI falls within the range of the Z-score from −2 SD to 1 SD, underweight: <−2 SD to −3 SD, overweight: 1 SD to <2 SD, obesity from 2 SD to 3 SD [27]. As shown in the Table 2, in the control group, the mean BMI after 12 months is closely approaching being overweight (0.914) and the 3rd quartile of this parameter equals 1.434. This means that being overweight is popular in the control study, whereas the intervention group values fall within the normal weight range.

Both groups underwent blood tests at the beginning of the study and one year later. The comparison between the initial and final results of blood tests showed that the intervention group showed statistically significantly lower levels of TG, TG/HDL ratio parameters and higher concentration of albumins (Table 3). 

Comparing the delta values of blood parameters showed that statistically significant lower cholesterol and TG levels were observed in the intervention group (Table 4).

We also analyzed the measurements in terms of being within the reference ranges. The control group was characterized by a higher number of children with excessively high TG levels and TG/HDL ratio values while inadequate HDL levels (Table 5).

Finally, the diet composition and macronutrient content were estimated. After a one-year dietary education, children from the control group had a statistically higher dietary intake of energy, fats, carbohydrates, and saccharose and a lower intake of fiber (Table 6). In addition, in the control group, their diets were less varied than those of their peers in the intervention group, with significant amounts of sweets, processed food and ready-to-eat children’s food. Infants from the intervention group ate more vegetables and fruits, had various sources of complex carbohydrates (groats, pasta, rice) and complete protein in lean meat and dairy in their diets.

## 4. Discussion

To the best of the authors’ knowledge, our project is the first to evaluate the impact of parents’ education on children’s anthropometric-metabolic parameters at such an early age. The messages sent contained information on the general principles of infant nutrition, diet expansion and composition and appropriate reading of the signs of hunger and satiety. It is the first study to assume such an early start in nutritional education. Our study showed that parental nutritional education resulted in less increment of body mass and body mass index, with lower cholesterol, TG, TG/HDL ratio value, energy, fats, carbohydrates, and saccharose intake in the intervention group. In addition, the control group had lower HDL, albumins parameters and lower fiber intake.

Different studies have already shown the positive effects of nutritional education on weight in children [15,16,17]. However, our study is the first in Central and Eastern Europe to start education for children at this early age and engage both parents in a very simple and low-cost way using modern technology. Our study included a specific group of patients in the nutrition of which, there are many limitations. On the other hand, the obtained effect may permanently program the child’s metabolism. 

A recently conducted study in Norway showed that children of low-educated parents gained more excess weight at two years than children of high-educated parents (total effect, RRTE = 1.06; 95% CI 1.01, 1.10) [16]. Furthermore, children of low-educated parents were more likely to be overweight or have obesity than those of high-educated parents [16].

A five-week nutritional intervention for parents of overweight children and children with obesity at the age of 7–12 years old proved the effectiveness of the parent involvement intervention in promoting the child-parent relationships and dietary self-efficacy of children [17]. However, the authors of the study stated that a five-week intervention was insufficient to produce significant changes in children’s body mass index [17]. Our study not only included children at such a young age but also provided long-term nutritional education and nutritional consultation to parents. Thus, we were able to receive significant differences in body mass between the groups.

It is worth noting that most studies are conducted on children who already have obesity or are overweight [15]. Thus, the intervention focuses primarily on weight loss and lifestyle changes. Our study assumed prevention. Adequate introduction and amounts of complementary food are required to meet the correct supply of macronutrients in children’s diets. In this research, mothers learnt how to appropriately expand their children’s diet. They received detailed guidelines on the correct time to introduce complementary meals, the sequence of introducing new products and ideas for meals. Important information for mothers was the appropriate amount of nutrition that the baby should receive and the frequency of serving meals. The acquired knowledge was implemented in the diets of children. Children in the intervention group consumed more vegetables and fruits than children in the control group and their diets were more varied. The nutrition of the control group was mainly based on products intended for children, including a large supply of modified milk and porridges for infants, as well as sweets and processed food.

Prevention of diseases is always more advisable than disease treatment. In 2013, Ruiter et al. found that 61.0% of parents in the Netherlands underestimated the weight status of their overweight children aged 2–12 years [34]. The parents’ underestimation of their children’s overweight/obesity status was associated with the children’s age (the age of two to seven years; OR: 0.18), gender (most common in males; OR: 0.55) and the parents’ education level (more often in parents with middle and high education; OR: 0.56 and 0.44, respectively) [34]. Consequently, parents’ underestimation of their children’s weight status remained alarmingly high, particularly among parents of young, children with obesity [34]. Furthermore, Jaballas et al. proved that 40% of parents of third-grade children in urban schools believed that being overweight is a condition that will be outgrown [35]. Parents who ate more meals than recommended tended to underestimate their children’s excessive body weight [35]. Prevention should also include a regular anthropometric evaluation by health specialists, as parents’ perception of their children’s body weight is biased [36]. Furthermore, parents struggle with helping their children in the process of losing weight [37]. In addition to not recognizing the problem, parents struggle with finding enough time and appropriate means to encourage the change [37,38]. Schalkwijk et al. discovered that parents of children with obesity struggled with adopting and adhering to new dietary rules, while the children struggled with inconsistent parenting and a lack of parental support [39]. First, parents need to have nutritional knowledge, proper nutritional habits and support from the health specialists and reliable guidelines [39,40]. It is worth emphasizing that our study perfectly meets the needs mentioned above of parents, providing them with long-term and substantive support in the field of nutrition for their children.

According to Cislak et al., a lack of restrictive control over food choices, a high intake of healthy foods and a low intake of unhealthy foods by parents and siblings, and a low pressure to consume foods support the development of healthy eating habits in children [41]. Gouveia et al. proved that mothers of overweight children or children with obesity had lower levels of emotional awareness of their child, non-judgmental acceptance of parental functioning, and pushed their children to eat [42]. In our study, parents received guidelines on not only healthy infant nutrition but also on the importance of eating meals together as a family and setting a good example for their children. The availability of direct contact with a dietitian encourages parents to ask questions and address their concerns. Thus, parents were more eager to provide solid foods to their children and trust them with their hunger and satiety cues.

Previous experiences (i.e., the winter of ‘the Great Hunger’) have demonstrated that improper nutrition in the first 1000 days can lead to atherosclerosis, hypertension, and other metabolic diseases [19,20]. Although the concentration of total cholesterol and its fraction did not differ statistically significantly in both groups, the increase in cholesterol concentration was greater in the control group. On the one hand, it is difficult to anticipate substantial changes in the level of lipid metabolism in children at this age. On the other hand, interestingly, the TG/HDL ratio (a parameter associated with the risk of cardiovascular diseases and insulin resistance) was found to be higher in the control group than in the study group.TG/HDL ratio is a risk factor for cardiovascular diseases and insulin resistance. A cut-off point of 2.0 was established as a sign of elevated cardiovascular risk in adults [28]. Unfortunately, there are not any clear values ranges for children. In De La Cruz Ruiz-Jaramillo and López-Acevedo’s study, the median TG/HDL index was 2.0 for children with obesity (7 ± 2 years) without metabolic syndrome [29]. Olson et al. study showed that children with TG/HDL ratio ≥3 were heavier and had higher blood pressure, glucose, HOMA-IR, LDL number, lower HDL level, QUICKI, and LDL size [43]. Taking these studies into consideration, we established a cut-off point to be 2.0 since it concerned healthy infants. With this cut-off point, up to 41% of children in the intervention group and only 2% of children in the control group would not be at risk of developing cardiovascular diseases. The fact that up to 98% of infants in the control group had a TG/HDL ratio above the cut-off point can be attributed to parents’ reluctance to feed their infants vegetables and fruit and prefer to keep them on animal foods (sweetmeat, porridge in milk). Such eating behavior can also suppress the higher levels of TG found in this group of infants in our study.

## 5. The Limitations of the Study and Implications for Further Research

Recruiting parents of infants in Outpatient Pediatric Clinics influenced the group’s diversity. Most of the parents came from villages within urban agglomerations. This fact also influenced education—the vast majority of parents had higher education. Higher education may result in a higher nutritional education level at the baseline. It is crucial to notice that despite widespread dietary knowledge and education, parents still make many mistakes in infants’ nutrition. The educational program in this study did not differ in parental education. The positive effect of the intervention was observed regardless of the level of parental education at the baseline. Parents are always in doubt when it comes to filling out the nutrition diary. Before filling out the diaries, parents were trained by a dietitian. In addition, most of them were college-educated.

It is also doubtful about the fact that only parents are responsible for feeding their children. Also, other closest members of the rest of the family influence children’s eating. Nevertheless, in an interview with a dietitian, parents indicated people who were responsible for their children’s nutrition. In 85% of cases, it was only the mother, in 5%-the father, and in 10%-both parents.

The limitation of the study is the evaluation of the short-term effects of education. We would like to re-check the groups in a few years (e.g., 5, 10 years) to see how the parents’ education in early childhood influenced their later health.

The basis for further research should be enrolling parents from different regions across the country. Moreover, the parents’ income levels should be considered to understand the target population. This study’s lack of analysis of parents’ earnings is a limitation. What is more, it is worth evaluating (after 5–10 years) whether or not early nutritional education adds to the beneficial nutritional status in later life.

## 6. Conclusions

Our study showed that parental nutritional education influences, among others, the BMI Z-score (the difference between the groups was 1.039) and the TG/HDL ratio (*p* < 0.001) in children. The final results of our study showed that proper nutritional education could improve children’s nutritional status at the population level. The parents’ knowledge of the principles of children’s nutrition would enhance the right child development by protecting it from metabolic disorders and their implications in the future. We have an opportunity to develop and implement innovative methods of educating parents at a very low cost on a broader scale. It can be easily implemented across the whole region or country. Thus, it is crucial to cover the nutritional program for parents.

## Figures and Tables

**Table 1:** Data concerning parents’ age, education, and place of residence.

Parameters	Parents GR 1	Parents GR 2	*p*
Age (years) ^1^	30 (28–34)	30 (28–34.5)	ns
Place of residence ^1^			ns
Village (from the city agglomeration)	63%	58%	
a city with fewer than 500 thousand residents	26%	30%	
a city with more than 500 thousand residents	11%	12%	
Education ^1^			ns
Primary	0%	3%	
Secondary	25%	20%	
Higher	75%	77%	

GR 1, intervention group; GR 2, control group; value, Median (1st–3rd quartile)/%; ^1^ Mann-Whitney test; ns, not significant.

**Table 2:** Body weight in infants.

Parameters	GR 1 (*n* = 80)	GR 2 (*n* = 80)	*p*
	**Median (1st–3rd quartile)**		
Birth weight (g) ^1^	3500 (3300–3710)	3598 (3170–3835)	ns
Z-score for birth weight ^1^	0.686 (0.294–1.098)	0.878 (0.039–1.343)	ns
Z-score for BMI ^1^	0.784 (0.312–1.123)	0.912 (0.052–1.434)	ns
ΔWeight after 12 months (g) ^1^	6285 (5730–6630)	7550 (6300–8400)	<0.001
	**Mean (1st–3rd quartile; SD)**		
Weight after 12 months (g) ^2^	9730 (9200–10,200; 0.540)	112,300 (10,100–12,000; 1.307)	<0.001
Z-score for weight after 12 months ^2^	−0.130 (−0.376–0.043; 0.274)	0.914 (0.144–1.458; 1.004)	0.004
Z-score for BMI after 12 months ^2^	−0.180 (−0.364–0.035; 0.204)	0.859 (0.132–1.372; 0.949)	<0.001

GR 1, intervention group; GR 2, control group; ^1^ Mann-Whitney test; ^2^ Student’s *t*-test; SD, standard deviation; ns, not significant.

**Table 3:** Comparison of metabolic parameters between the study groups at the end of the trial—end versus end.

Parameters	GR 1 (*n* = 80)	GR 2 (*n* = 80)	*p*
**Median (1st–3rd quartile)**			
Cholesterol (mg/dL) ^1^	163 (135–169)	157 (132–171)	ns
TG (mg/dL) ^1^	109 (93–127)	130 (108–166)	<0.001
HDL (mg/dL) ^1^	43 (39–50)	42 (35–47)	ns
LDL (mg/dL) ^1^	93 (73–101)	84 (64–100)	ns
TG/HDL ratio ^1^	2.52 (1.68–2.96)	3.10 (2.88–4.07)	<0.001
Glucose (mg/dL) ^1^	81 (76–86)	80 (68–88)	ns
Protein (g/dL) ^1^	7.02 (6.67–7.10)	7.05 (6.94–7.12)	ns
Albumins (g/dL) ^1^	4.66 (4.43–4.75)	4.59 (4.29–4.76)	0.007

GR 1, intervention group; GR 2, control group; ^1^ Mann-Whitney test; cholesterol (normal value for females and males: 60–190); TG, triglycerides (normal value for females and males: <200 mg/dL); HDL, high-density lipoprotein (normal value for females and males: >35 mg/dL); LDL, low-density lipoprotein (normal value for females and males: <155 mg/dL); TG/HDL ratio (recommended value for females and males <2.0); glucose (normal value for females and males: 60–100 mg/dL); protein (normal value for females: 7.04–7.46 g/dL and males: 7.12–7.50 g/dL); albumins (normal value for females and males: 4.16–4.72 g/dL) [28,29,32,33]; ns, not significant.

**Table 4:** Comparison of metabolic parameters between the study groups at the end of the trial-delta versus delta.

Parameters	GR 1 (*n* = 80)	GR 2 (*n* = 80)	*p*
**Median (1st–3rd quartile)**			
ΔCholesterol (mg/dL) ^1^	8 (−20–23)	22 (−20.5–52)	0.002
ΔTG (mg/dL) ^1^	−53 (−81–27)	−14 (−62–38)	0.001
ΔHDL (mg/dL) ^1^	−0.7 (−3.3–3.4)	−2.3 (−22.2–7.3)	ns
ΔLD (mg/dL)L ^1^	31.8 (−18.2–39.7)	14.5 (−6–51.7)	ns
ΔGlucose (mg/dL) ^1^	−2 (−14–14)	3 (−8.5–15.5)	ns
ΔProtein (g/dL) ^1^	1.21 (0.61–1.89)	1.92 (0.51–2.02)	ns
ΔAlbumins (g/dL) ^1^	0.37 (0.35–1.18)	0.82 (0.23–1.13)	ns

GR 1, intervention group; GR 2, control group; ^1^ Mann-Whitney test; cholesterol (normal value for females and males: 60–190); TG, triglycerides (normal value for females and males: <200 mg/dL); HDL, high-density lipoprotein (normal value for females and males: >35 mg/dL); LDL, low-density lipoprotein (normal value for females and males: <155 mg/dL); glucose (normal value for females and males: 60–100 mg/dL); protein (normal value for females: 7.04–7.46 g/dL and males: 7.12–7.50 g/dL); albumins (normal value for females and males: 4.16–4.72 g/dL) [28,29,32,33]; ns, not significant.

**Table 5:** Comparison of metabolic parameters between the study groups at the end of the trial.

Parameters	GR 1 (*n* = 80)		GR 2 (*n* = 80)		*p*
	High	Norm	High	Norm	
Cholesterol (mg/dL) ^1^	16	64	20	60	ns
TG (mg/dL) ^1^	53	27	70	10	0.002
LDL (mg/dL) ^1^	3	77	0	80	ns
Glucose (mg/dL) ^1^	0	80	0	80	ns
	Low	Norm	Low	Norm	
HDL (mg/dL) ^1^	6	74	20	60	0.005
Protein (g/dL) ^1^	15	65	23	57	ns
Albumins (g/dL) ^1^	29	51	30	50	ns
	High	Norm	High	Norm	
TG/HDL ratio	47	33	78	2	<0.001

GR 1, intervention group; GR 2, control group; ^1^ Chi^2^ test; cholesterol (normal value for females and males: 60–190); TG, triglycerides (normal value for females and males: <200 mg/dL); HDL, high-density lipoprotein (normal value for females and males: >35 mg/dL); LDL, low-density lipoprotein (normal value for females and males: <155 mg/dL); TG/HDL ratio (recommended value for females and males <2.0); glucose (normal value for females and males: 60–100 mg/dL); protein (normal value for females: 7.04–7.46 g/dL and males: 7.12–7.50 g/dL); albumins (normal value for females and males: 4.16–4.72 g/dL) [28,29,32,33]; ns, not significant.

**Table 6:** Dietary intake in children.

Dietary Intake (% RDA)	GR 1 (*n* = 80)	GR 2 (*n* = 80)	*p*
	**Median (1st–3rd quartile)**		
Energy ^1^	108.97 (103.75–113.32)	132.12 (123.73–143.25)	<0.001
Proteins ^1^	313.080 (283.780–334.520)	305.910 (265.460–401.880)	ns
Fats ^1^	90.76 (87.44–110.03)	109.69 (94.07–132.51)	<0.001
Carbohydrates ^1^	101.00 (95.96–108.39)	124.59 (113.22–141.66)	0.029
Saccharose ^1^	9.47 (8.68–11.64)	13.81 (5.39–16.82)	<0.001
Fiber ^1^	130.900 (103.100–179.750)	82.000 (63.900–99.000)	<0.001

GR 1, intervention group; GR 2, control group; RDA, recommended dietary allowance according to Polish nutritional standards [31]; ^1^ Mann-Whitney test; ns, not significant.

## Data Availability

The data presented in this study are available on request from the corresponding author.
